# New insights of the local immune response against both fertile and infertile hydatid cysts

**DOI:** 10.1371/journal.pone.0211542

**Published:** 2019-01-30

**Authors:** Christian Hidalgo, Caroll Stoore, Karen Strull, Carmen Franco, Felipe Corrêa, Mauricio Jiménez, Marcela Hernández, Karina Lorenzatto, Henrique B. Ferreira, Norbel Galanti, Rodolfo Paredes

**Affiliations:** 1 Escuela de Medicina Veterinaria, Facultad de Ciencias de la Vida, Universidad Andres Bello, Santiago, Chile; 2 Staff pathologist, Clinica Santa Maria, Santiago, Chile; 3 Laboratorio de Biología Periodontal, Facultad de Odontología Universidad de Chile, Santiago, Chile; 4 Facultad de Ciencias de la Salud, Universidad Autónoma de Chile, Santiago, Chile; 5 Laboratório de Genômica Estrutural e Funcional, and Laboratório de Biologia Molecular de Cestódeos, Centro de Biotecnologia, UFRGS, Porto Alegre, Rio Grande do Sul, Brazil; 6 Programa de Biología Celular y Molecular, Instituto de Ciencias Biomédicas, Facultad de Medicina, Universidad de Chile, Santiago, Chile; Seconda Universita degli Studi di Napoli, ITALY

## Abstract

**Background:**

Cystic echinococcosis is caused by the metacestode of the zoonotic flatworm *Echinococcus granulosus*. Within the viscera of the intermediate host, the metacestode grows as a unilocular cyst known as hydatid cyst. This cyst is comprised of two layers of parasite origin: germinal and laminated layers, and one of host origin: the adventitial layer, that encapsulates the parasite. This adventitial layer is composed of collagen fibers, epithelioid cells, eosinophils and lymphocytes. To establish itself inside the host, the germinal layer produces the laminated layer, and to continue its life cycle, generates protoscoleces. Some cysts are unable to produce protoscoleces, and are defined as infertile cysts. The molecular mechanisms involved in cyst fertility are not clear, however, the host immune response could play a crucial role.

**Methodology/Principal findings:**

We collected hydatid cysts from both liver and lungs of slaughtered cattle, and histological sections of fertile, infertile and small hydatid cysts were stained with haematoxylin-eosin. A common feature observed in infertile cysts was the disorganization of the laminated layer by the infiltration of host immune cells. These infiltrating cells eventually destroy parts of laminated layer. Immunohistochemical analysis of both parasite and host antigens, identify these cells as cattle macrophages and are present inside the cysts associated to germinal layer.

**Conclusions/Significance:**

This is the first report that indicates to cell from immune system present in adventitial layer of infertile bovine hydatid cysts could disrupt the laminated layer, infiltrating and probably causing the infertility of cyst.

## Introduction

Cystic echinococcosis (CE) is a major zoonotic disease caused by infection with the metacestode stage (hydatid cyst) of the flatworm *Echinococcus granulosus*. It has a worldwide distribution with an estimated 4 million people infected and another 40 million at risk [1]. High parasite prevalence is found in Eurasia, Africa, Australia and South America. CE affects more severely South American countries characterized by extensive grazing livestock farming including Argentina, Brazil, Chile, Peru, and Uruguay [2]. The life cycle of this parasite includes two mammal hosts. The definitive hosts are dogs and other canids; while ungulates and other mammals act as intermediate hosts [3] such as sheep, goats, cattle, pigs, buffaloes, horses and camels [4]. The most common infection sites in cattle are the liver and lungs [5–7]. Within these viscera, a unilocular cyst forms, that will grow gradually from 1 to 5 cm a year [8]. The hydatid cyst is circumscribed by a layer generated by the intermediate host in response to the parasite, named adventitial layer, which mainly consists of epithelial cells and connective tissue [9]. The adventitial layer can have variable thickness and may present some focal fibrosis as result of host immune response that considers the cyst a foreign body [10]. The lumen of the hydatid cyst is filled with the so called hydatid fluid and is surrounded by two layers of parasite tissue; the innermost cellular layer is called germinal layer and is intimately attached to an acellular layer called laminated layer, the latter is in close contact with the adventitial layer. The germinal layer is composed by embryonic cells whose function is to elaborate the different elements of hydatid cysts [10]. These embryonic cells differentiate into buds that finally generate protoscoleces (PSC), the infectious parasite form for the definite host [8]. The laminated layer is generated by the germinal layer and is described as a specialized extracellular matrix that is found only in the genus *Echinococcus* [11]. Macroscopically, it is seen as a whitish membrane, formed by various layers of mucopolysaccharides and keratin, evolutionarily adapted to maintain the physical integrity of metacestodes and to protect the cells of the germinal layer from host immunity [12]. In the intermediate host, it is possible to find two different types of hydatid cysts: fertile hydatid cysts, in which PSC are attached to the germinal layer and free into the hydatid fluid. Fertile hydatid cyst PSC viability, that is, the percentage of live PSC, varies between 100% and 2,8% [13–17]. Contrarily, infertile hydatid cysts (also called sterile hydatid cysts [18–23]), have no PSC neither attached to the germinal layer nor floating free in the hydatid fluid, and thus are unable to continue with the parasite life cycle. The reason behind why infertile hydatid cysts are unable to produce PSC remains unclear [24]. In many geographical areas, including Chile [25], cattle has been associated with low fertile hydatid cysts counts (<30%) in both *Echinococcus granulosus sensu lato* [26–29] and *Echinococcus granulosus sensu stricto* [6, 22], so it is a suitable model to study cyst infertility mechanisms. Our research team, so far has been working in understanding the causes of infertile hydatid cyst in cattle, identifying both higher apoptosis levels in germinal layer of infertile cysts [24] and different immunoglobulin profiles [30]. Possible relations of the laminated and adventitial layers with fertility or infertility, however, have never been addressed. In this work, we present a systematic comparative study of both the laminated and adventitial layer in fertile and infertile hydatid cysts obtained from naturally infected cattle. Protoscolex viability of fertile hydatid cysts is determined; morphohistological characteristics of hydatid cysts are described and compared, demonstrating the infiltration of host immune cells inside infertile hydatid cysts, and providing evidences of their effect on and contribution to cyst integrity and fertility.

## Materials and methods

### Sample collection, classification and genotyping

All bovine hydatid cyst samples were obtained at an abattoir in Santiago, Chile, as part of the normal work of the abattoir and with consent from both the veterinarians and the owners of the abattoir for sample collection. Both lung and liver samples were manually inspected by the official health office veterinarian and afterwards by our research team. This protocol study was approved by the Universidad Andres Bello Bioethics Board (protocol number 016/2016). For each positive viscera, the hydatid cysts were removed and placed in a sealed plastic bag and were stored at 4°C.

In the laboratory, each hydatid cyst was assigned a number, the hydatid fluid was aseptically aspirated and the cyst was opened along the longer axial plane. Fertile hydatid cysts were classified as such when: 1.- The laminated and germinal layers were white and thick and easily detach from the adventitial layer; 2.- PSC were found both attached to the germinal layer and floating in the hydatid fluid. PSC viability was determined with trypan blue staining, and samples with viability lower than 85% were classified as low viability. Infertile hydatid cysts were classified as such when 1.- The laminated layer was yellow/ochre and thin, and tightly adhered to the adventitial layer; 2.- There were no visible PSC attached to the germinal layer nor floating in the hydatid fluid; 3.- a sample of the germinal layer was observed under a conventional light microscope to confirm the absence of PSC. Since smaller hydatid cysts (<1 cm in diameter) are still developing and have not started producing PSC, they were classified as “small cysts”. After the cyst fertility assessment, a sample of the cyst wall containing all three layers was fixated in Glyo-Fixx and embedded in paraffin. All samples were processed within 24 h of their procurement.

All hydatid cyst samples were genotyped using the method described by Bowles et al [31] in combination with sequencing of PCR products. Only samples from *Echinococcus granulosus* sensu stricto were used in this study.

### Haematoxylin-eosin morphohistological analysis

Paraffin blocks were cut to obtain 5 μm thickness sections and were stained with haematoxylin-eosin (H&E). Using an Olympus FSX100 microscope, each slide was examined to confirm that the three hydatid cyst layers were present. All infertile and small cyst samples that lacked the laminated layer were excluded from the analysis, however, all fertile cysts were included due to the small sample size.

A seasoned pathologist examined blindly each slide, describing the germinal, laminated and adventitial layer features. For the adventitial layer, a score index was assigned to the overall inflammation present in the tissue, as well as scores for Lymphocytes, Plasmatic Cells, Fibroblasts, Macrophages, Giant Multinucleated Cells and Eosinophils, grading them with a score from 0 to 3 according to the following criteria: Mild (1) up to 30 inflammatory cells per high power field (HPF); Moderate (2) 30 to 100 inflammatory cells per HPF; Severe (3) more than 100 inflammatory cells per HPF. Assessment was done in an average of 10 HPF. Host immune cells were identified by the pathologist based on their morphology and staining pattern in H&E. For statistical analysis, inflammation scores were divided between low (0.5 to 1.5) and high (2 to 3).

The thickness of the laminated layer was evaluated with the FSX-BSW software package that is included with the FSX100 microscope. The laminated layer length was measured in 20 consecutive areas in μm, obtaining both an average and its standard deviation.

For statistical analysis, Data was recorded in Excel 2010 datasheet and analyzed using RStudio IDE version 1.0.136 with R version 3.3.3. Outliers were identified using ROUT method with Q = 1% and differences were calculated using a two-way ANOVA with Tuckey’s post hoc analysis for quantitative variables. Chi squared test was applied to compare differences between categorical variables. Statistical significance was considered with a P-Value <0.05.

### Immunohistochemical (IHC) analysis

To differentiate between parasite and host cells, we used two antibodies: one that targets *Echinoccocus granulosus* aldolase (EgAldo) and another that targets host macrophages (Invitrogen S100A9 Monoclonal Antibody [MAC387]). Briefly, paraffin blocks were cut to obtain 3 μm thickness sections, and after deparaffinization and rehydration, for EgAldo, antigen retrieval was performed with Citrate Buffer (10 mM sodium citrate, 0,05% Tween-20, pH 6.2), the primary antibody was incubated overnight at 4°C at a dilution of 1:1000. For cattle macrophages, antigen retrieval was performed by incubating slides with a 0.05% trypsin solution at 37°C for 15 minutes, the primary antibody was incubated 1 hour at room temperature at a dilution of 1:200. HRP-Conjugated secondary antibody (Jackson ImmunoResearch) anti-Rabbit (EgAldo) or anti-mouse (cattle macrophages) was incubated for 1 h at room temperature at a dilution of 1:1000. Finally, DAB-Plus Substrate Kit (Life Technologies) was used for detection and slides were counterstained with haematoxylin.

## Results

### Bovine hydatid cyst sample distribution

From 2,961 bovines examined, 558 were positive to *Echinococcus granulosus* infection. Of these, 23 were fertile hydatid cyst, with PSC viability average of 43% and median of 50%. After the paraffin embedding process and microscopic evaluation, 144 samples were obtained with the three layers in succession; 2 fertile hydatid cysts with PSC viability >85% (1 in lungs, 1 in liver), 7 fertile hydatid cysts with low PSC viability (<85%) (3 in lungs, 4 in liver), 129 infertile (91 in lungs, 38 in liver) and 16 small cysts (10 in lungs, 6 in liver).

### The laminated layer thickness varies according to cyst location and type

**Fig 1:**
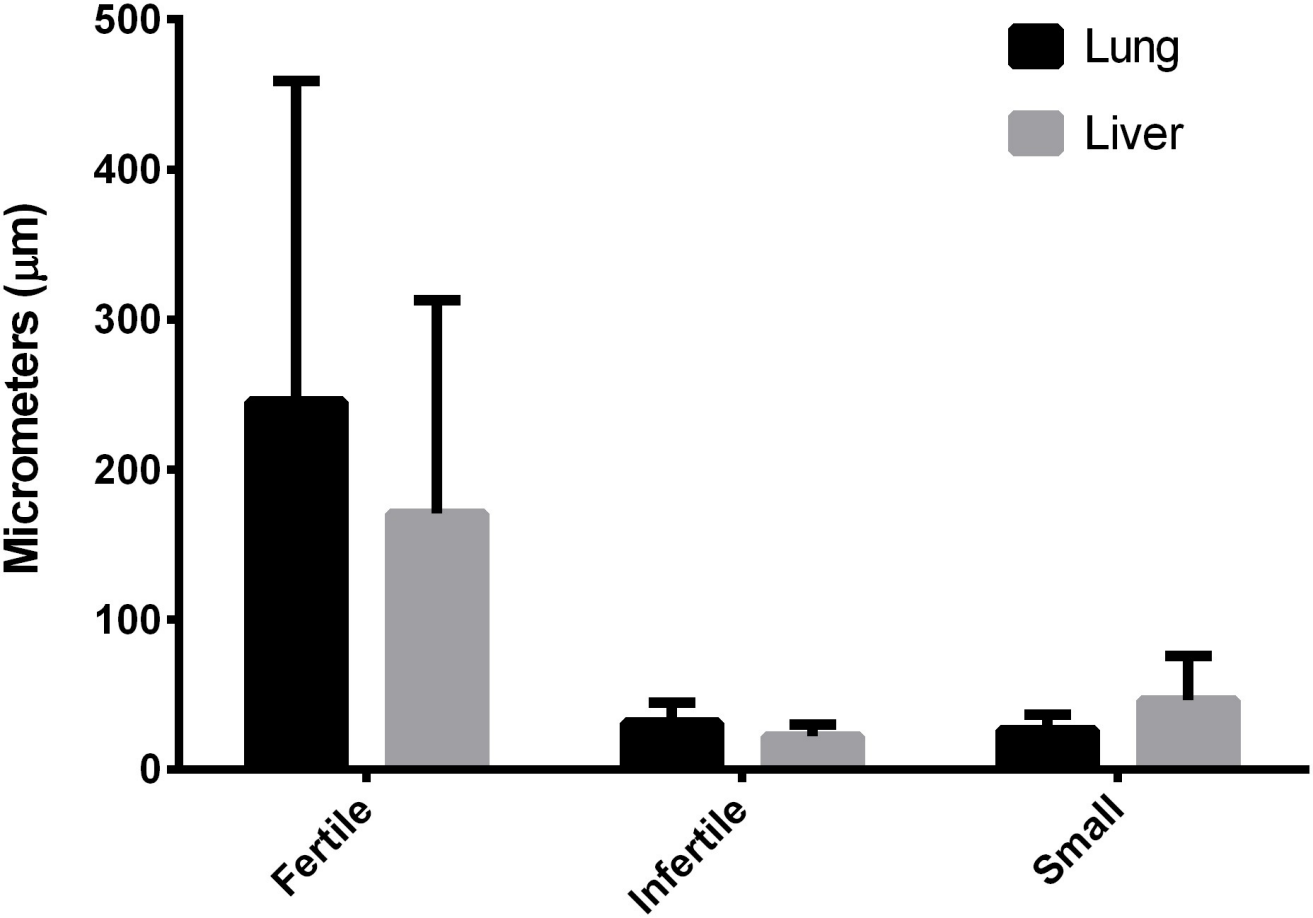
Laminated layer thickness (μm) according to hydatid cyst location and fertility. Data is shown as mean +/- standard deviation. Fertile, lung: n = 4. Fertile, liver: n = 5. Infertile, lung: n = 83. Infertile, liver: n = 36. Small, lung: n = 10. Small, liver: n = 6. The only statistical differences were between fertile and infertile/small cysts, regardless of organ localization.

Gross hydatid cyst examination of the laminated layer between fertile and infertile hydatid cysts was verified with microscopic measurement; fertile hydatid cysts have almost a six-fold thicker laminated layer than infertile and small hydatid cysts, 217 μm (± 18,4 μm) vs 33 μm (± 5,4), this difference is statistically significant (p <0.05). Conversely, small hydatid cysts have a laminated layer thickness similar to infertile hydatid cysts. There are also differences in the laminated layer thickness according to hydatid cyst location; whereas fertile and infertile hydatid cysts have overall thicker laminated layers when found in the lungs (36 μm in lungs vs 29 μm in livers), small cysts have thicker laminated layers when found in the liver (26 μm in lungs vs 49 μm in liver), these differences although, are not statistically significant (p > 0.05) (Fig 1). The complete laminated layer measurements are available as S1 Table.

No other statistically significant differences were found between lungs and liver cysts, so future results of fertile, infertile and small hydatid cysts are from both lungs and liver samples.

### The laminated layer disorganizes and is infiltrated by the adventitial layer

**Fig 2:**
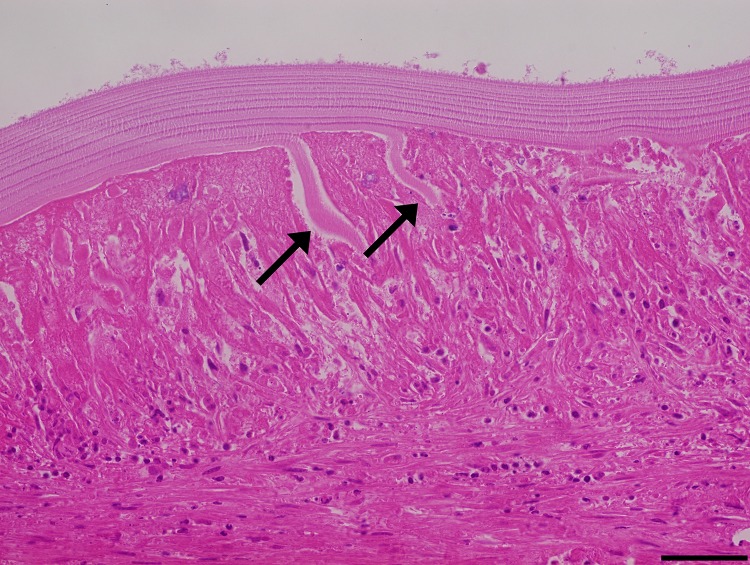
Laminated layer disorganization. The different layers that compose the laminated layer disorganize (arrows), while the adventitial layer inflammatory cells infiltrate between them. Size bar: 50 μm.

Histological sections of hydatid cyst wall tissue sections, reveals that the adventitial layer infiltrates the laminated layer. This is visualized as a disorganization of the layers that compose the laminated layer, with host cells in between. A representative image of this feature is shown in Fig 2. Also, whole sections of the laminated layer can be found within the adventitial layer while in other samples the difference between laminated and adventitial layer becomes difficult to establish (S1 Fig).

### Fertile, infertile and small hydatid cysts present hallmark histological features in the adventitial layer

**Fig 3:**
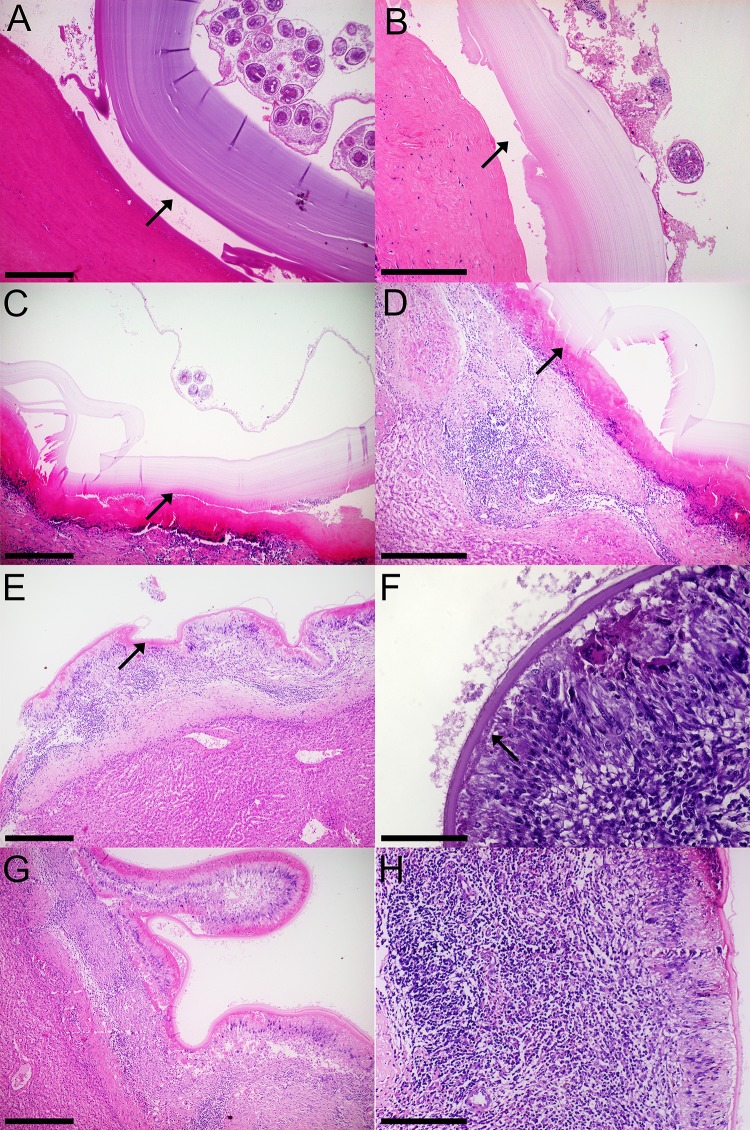
Histological characteristics of fertile, infertile and small bovine hydatid cysts from both lungs and livers. A, B: Fertile hydatid cyst with high viability protoscoleces (PSC) are characterized by a germinal layer with PSC, followed by a thick laminated layer and an adventitial layer composed of collagen fibers that detach from the laminated layer (arrows). C, D: Fertile hydatid cysts with low viability PSC feature the same germinal and laminated layer characteristic of high viability fertile hydatid cysts, but have an adventitial layer composed of inflammatory cells that are tightly attached to the laminated layer (arrows). E, F: Infertile hydatid cysts have little to none germinal layer cells with a thin laminated layer, the adventitial layer is composed palisading foamy macrophages, multinucleated giant cells and lymphoid follicles. This inflammatory infiltrate is tightly attached to the laminated layer (arrows). G, H: Small hydatid cysts (i.e. <1 cm) feature the same histological characteristics as infertile cysts. Size bar: A: 400 μm, B: 200 μm, C: 400 μm, D: 200 μm, E: 400 μm, F: 100 μm, G: 400 μm, H: 200 μm.

Fertile hydatid cysts with high PSC viability, have germinal layer with PSC attached, cells in different developmental stages and thick laminated layers (>100 μm) that easily detach from the adventitial layer (Fig 3A); this adventitial layer is composed mainly of collagen fibers and fibroblasts (Fig 3B). Inflammatory cells, when present, are found beneath the collagen and fibroblast layer. Fertile hydatid cysts that have low PSC viability, have thinner laminated layers (<100 μm), and while the collagen and fibroblast layer is present in the adventitial layer, the inflammatory cells are found beneath the laminated layer (Fig 3C and 3D). Conversely, infertile hydatid cysts all share common features: a thin laminated layer, sometimes thinner than 5 μm. Beneath the laminated layer, all infertile cysts have palisading foamy macrophages. Supporting these macrophages, there are lymphoid follicles and multinucleated giant cells throughout the adventitial layer, with little presence of collagen fibers or fibroblasts (Fig 3E and 3F). Likewise, small hydatid cysts share the same histological features of infertile cysts (Fig 3G and 3H).

### Inflammatory cell composition of the adventitial layer in fertile, infertile and small hydatid cysts

**Fig 4:**
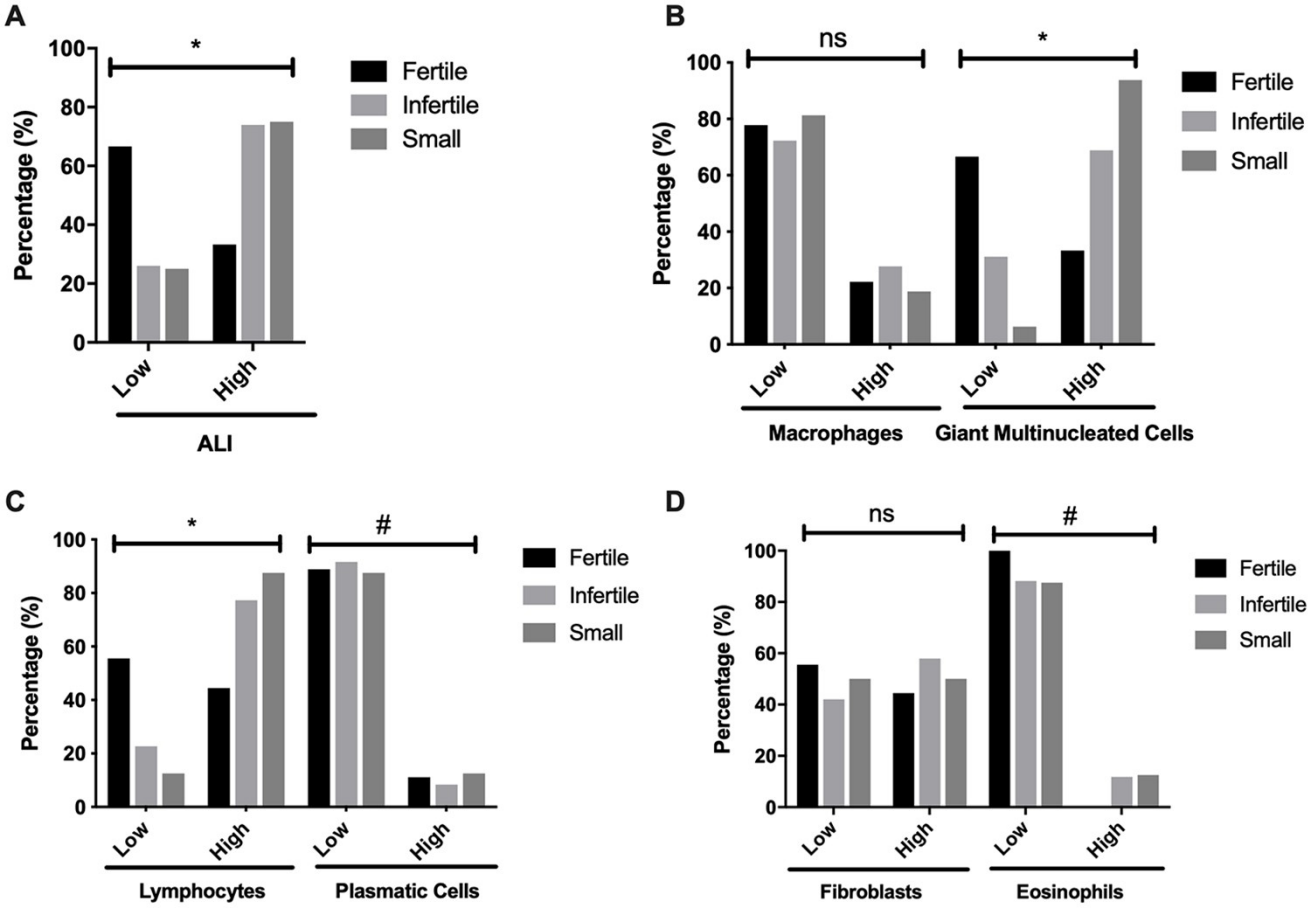
Inflammatory cell score between fertile (A), infertile (B) and small (C) bovine hydatid cysts from both lungs and liver. Cell score ranged from 0 to 3 and was divided between low (0 to 1) and high (2 to 3) scores. Data is presented as a percentage of total samples. ALI = Adventitial layer inflammation; GMC = Multinucleated giant cells. Fertile n = 9, Infertile n = 129, Small n = 16.

All hydatid cyst samples had immune cells in the adventitial layer; however, the magnitude and presence inflammation is different. Fertile hydatid cysts had low adventitial layer inflammation scores, with relative high numbers of lymphocytes and fibroblast. None of the fertile hydatid cysts had high infiltration with eosinophils. On the contrary, infertile hydatid cysts had more than 50% of the samples with high adventitial layer inflammation scores. High lymphocyte infiltration was present in less than 80% of the samples, and high infiltration of giant multinucleated cells present in more than 60% of the samples. Small hydatid cysts follow the same pattern, with high adventitial layer inflammation scores in more than 70% of the samples, with high infiltration of lymphocyte and giant multinucleated cells in more than 80% of the samples (Fig 4). Total inflammatory cells and specially lymphocytes and multinucleated giant cells were significantly higher in the infertile and small cysts when compared fertile ones (p<0.05). Raw inflammation score data is available as S2 Table.

### Infertile and small hydatid cysts present host immune cells inside the germinal layer

**Fig 5:**
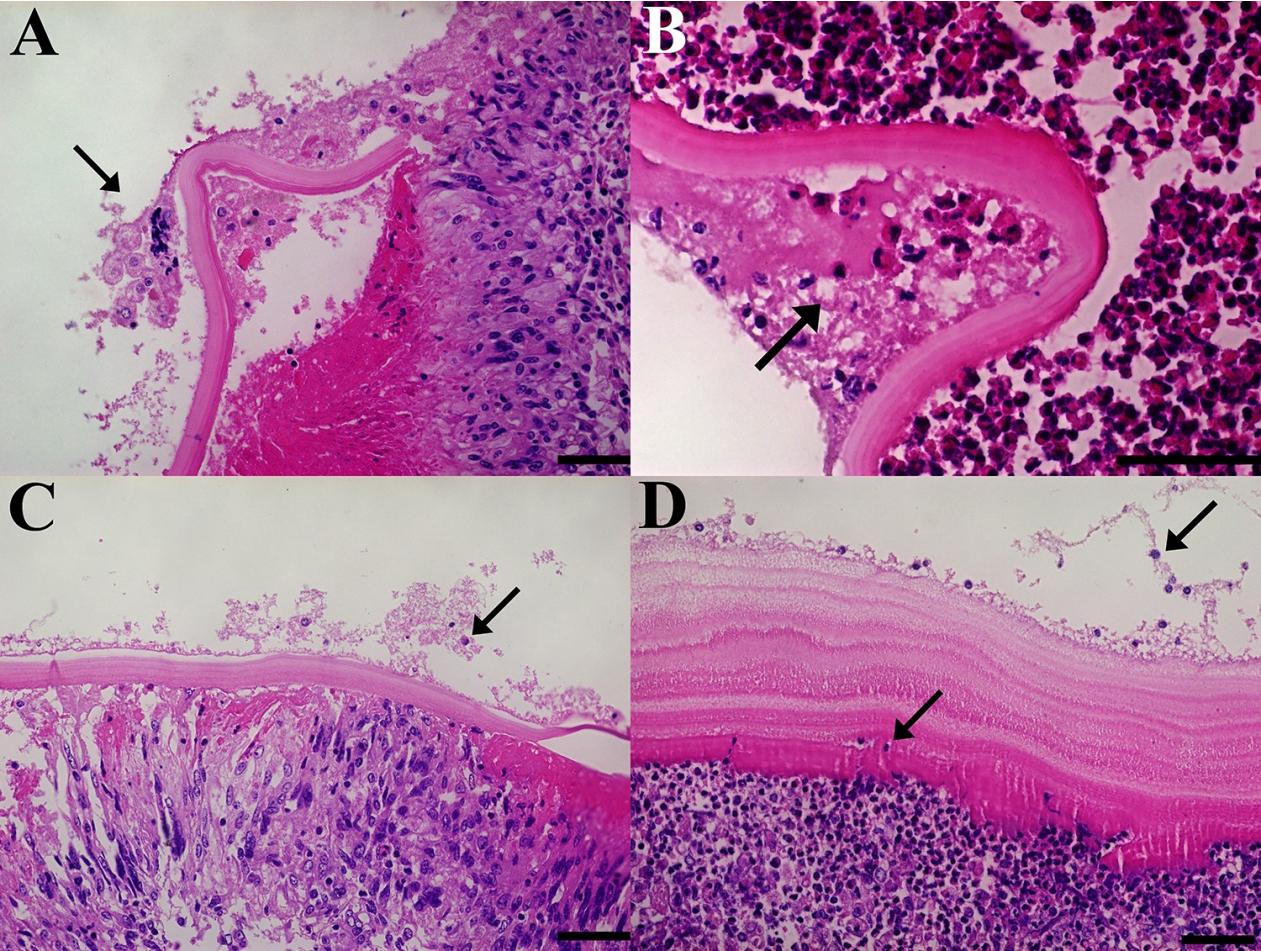
Cells with big nuclei within the germinal layer of both infertile and small hydatid cysts. Histological sections of cyst wall samples with the germinal, laminated and adventitial layers in succession. The arrow points at cells with bigger nuclei than parasite cells, either inside the laminated layer or within the germinal layer. Stained with H&E. Size bar: 50 μm.

Morphohistological analysis of both infertile and small hydatid cysts, revealed that between the germinal layer cells, there are many cells that have bigger nuclei than the small pignotic nuclei of *Echinococcus granulosus* germinal layer cells, suggesting a mammalian origin rather than parasite tissue. This feature was absent in fertile hydatid cysts, regardless of PSC viability. Cells with big nuclei could be found as big sheets (Fig 5A and 5B), as single cells (Fig 5C), and in some cases within both the laminated and germinal layers (Fig 5D). Morphologically, these cells resemble macrophages. It is worth noting that this finding is not present in every infertile hydatid cyst sample studied, so there are infiltrated and non-infiltrated infertile hydatid cysts. More images of how these cells are found in the germinal layer are available in S2 Fig.

**Fig 6:**
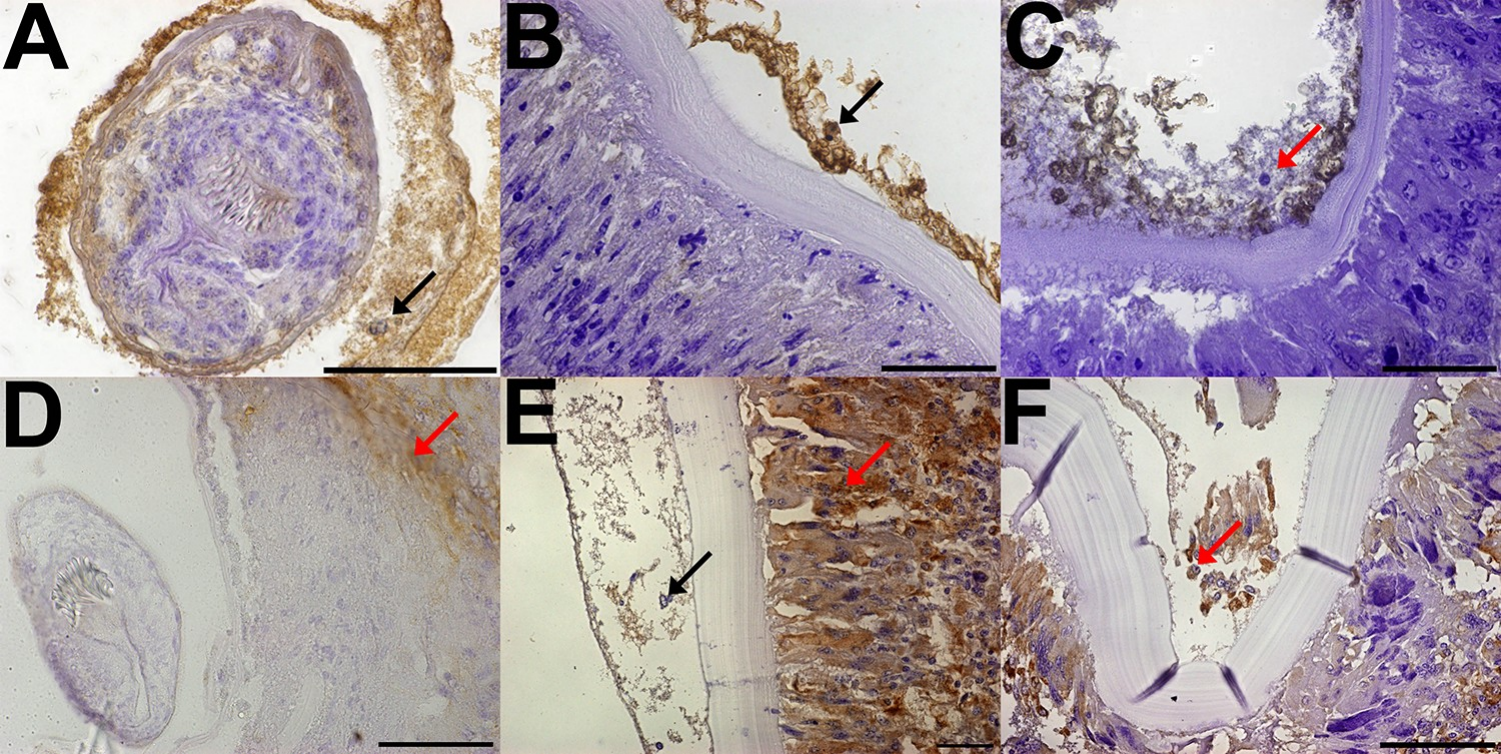
Immunohistochemical analysis of both fertile and infertile bovine hydatid cysts with anti-*Echinococcus granulosus* aldolase (A, B, C) and anti-macrophages antibodies (D, E, F). A: Fertile hydatid cyst, black arrow points at positive parasite cells inside the protoscolex. B: Infertile hydatid cyst without host cell infiltration, black arrow points at positive parasite cells along the germinal layer. C: Infertile hydatid cyst with host cell infiltration, red arrows points at host cells nucleus, which are negative to *Echinococcus granulosus* aldolase antibody detection. D Fertile hydatid cyst, red arrow points at positive macrophage while *Echinococcus granulosus* cells are negative E: Infertile hydatid cysts without host cell infiltration, red arrows points at positive macrophages while black arrow points at negative germinal layer cells. F: Infertile hydatid cyst with host cell infiltration, red arrow points at positive macrophages within both the germinal layer and the adventitial layer. Size bar: A, B, C, D, E: 50 μm. F: 100 μm.

To confirm the host origin of these cells, IHC analysis of fertile, and both infiltrated and non-infiltrated infertile hydatid cysts, using EgAldo antibody for parasite cells and Macrophage marker for host cells, demonstrates that these cells are not of parasite origin, as EgAldo is strongly positive in PSC (Fig 6A) and the germinal layer of non-infiltrated infertile hydatid cyst (Fig 6B), and is only partially positive in the germinal layer of infiltrated infertile hydatid cysts, with negative detection in the cytoplasm of big nuclei cells (Fig 6C); this in conjunction with the macrophage marker, that is negative in both PSC (Fig 6D) and germinal layer of non-infiltrated infertile hydatd cyst (Fig 6E), while strongly positive in the adventitial layer (Fig 6E and 6F) as well as in the germinal layer of infiltrated infertile hydatid cysts (Fig 6F).

## Discussion

*Echinococcus granulosus* infection of the intermediate host elicits a granulomatous tissue reaction; characterized by the accumulation of cells of monocytic origin and are thought to be directed both at walling off and at eliminating the persistent foreign body. The hallmarks of granulomatous reactions are special types of activated macrophages called epithelioid cells and multinucleated giant cells [32].

The adventitial layer is usually described as a fibrous layer due to the host’s reaction to the parasite [9, 33–39], and several studies have described the cell composition of this layer. A study with fertile hydatid cysts found in the liver, showed that the adventitial layer has a significant amount of B lymphocytes, occasional polymorphonuclear cells and monocytes, however, they did no correlate this data with PSC viability, as the study was done in formalin-fixed, paraffin-embedded tissue samples [33]. Another study compared the differences in the adventitial layer between ovine and macropod hydatid cysts, with the adventitial layer of fertile hydatid cysts consisting of palisading macrophages with foamy cytoplasm and multinucleated giant cells or with granulation-type fibrous tissue devoid of a discernable covering epithelial layer [35]; and although the authors ponder whether hydatid cysts will develop and become fertile under inflammatory conditions, they do not correlate PSC viability with the characteristics they describe. The most complete study of the adventitial layer of bovine hydatid cysts was done by Sakamoto and Cabrera [9], where they describe that infertile cysts have lymphocytes, macrophages, granulocytes and polynuclear giant cells infiltrating the adventitial layer, with the smaller infertile cysts being surrounded by macrophage derived cells while eosinophils are involved in the response against larger hydatid cysts, and although they did exhaustive IHC analysis of the adventitial layer, surprisingly they did not report positive staining in the germinal layer of infertile hydatid cysts. Our results expand on the characteristics of the adventitial layer of hydatid cysts, adding to the cellular infiltrate the presence of disorganization of the laminated layer and infiltration of adventitial layer cells, a feature not reported by the previous authors. Also we have a correlation between PSC viability and the overall inflammatory infiltrate in the adventitial layer of fertile hydatid cysts, and found that low viability fertile hydatid cysts correlate with higher inflammatory infiltrates.

When comparing hydatid cysts from both liver and lungs, the thickness of the laminated layer was different between fertile and infertile hydatid cysts and also between liver and lungs tissue. It has been described that the parenchyma of these organs limits the growth rate of the hydatid cyst, with the lung being less dense that the liver [37], so it makes sense that the laminated layer is thicker in the lung cysts compared to liver cyst; small cysts on the other hand, had an inverse tendency; however, these differences were not statistically significant. No differences where found between the inflammatory reaction in the adventitial layer of either liver or lung hydatid cysts; as both organs have resident macrophages (Küpffer cells and interstitial macrophages, respectively [40]), the granulomatous reaction against the hydatid cyst could have similar mechanisms.

Although a fertile hydatid cyst is defined solely by the presence of PSC, we propose that a more complete definition should include viable PSC; as shown in this study, fertile hydatid cysts with low viability or dead PSC have adventitial layer characteristics of infertile hydatid cysts, and if sampled later, they possibly would be classified as such. However, because we work with natural infection samples, we are not able to confirm when the animals acquired the infection.

Many small hydatid cysts samples had adventitial layer characteristics found in both low viability fertile hydatid cysts and infertile hydatid cysts. There is evidence that cysts from the same parasite strain, in the same organ and host can have differences in size, viability and fertility [39]. After examining 16 small hydatid cysts, all of which had adventitial layers with a strong immune reaction, we propose that small hydatid cysts with adventitial layer featuring palisading foamy macrophages, lymphocytes arranged in follicles, multinucleated giant cells, thin (<50 μm) laminated layer and host immune cells inside the germinal layer, should be regarded as infertile; while small cysts without these characteristic histological features could develop into either fertile or infertile hydatid cysts. Moreover, small cysts as well as infertile ones, showed significantly higher inflammatory infiltrates, particularly of lymphocytes and multinucleated giant cells, suggesting that immune response is directly involved in the cyst viability.

The laminated layer, which is secreted by the parasite contains mucins with *O*-type glycosylations and inositol hexakisphosphate (InsP_6_); these features are related to the parasite survival inside large mammals, and it has been proposed that the laminated layer is involved in down-regulating the local inflammatory response [11]. Also, it has been shown in mice that both macrophages and dendritic cells are activated by portions of the laminated layer [41]. In our results, many infertile and small hydatid cysts samples have clear signs of the host immune cells infiltrating the laminated layer and disorganizing it; with the eosin staining being more intense were this is happening; this could be due to the host macrophages secreting a combination of cathepsin K [32] and MMP-9 [42], although further experiments are needed to corroborate this.

The presence of host immune cells in direct contact with the germinal layer of infertile hydatid cysts has not been described before. Both the hydatid fluid and germinal layer of both fertile and infertile hydatid cysts usually contain many proteins of host origin [1, 43]. How these proteins enter the hydatid cyst is not clearly defined; the germinal layer consists of a distal cytoplasmic syncytium from which microtriches project into the laminated layer; these two parasite structures form a barrier that deny access to both host defense macromolecules and cells [44]. Particles of the laminated layer are described to inhibit macrophage proliferation [45] and induce the production of arginase (inhibiting nitric oxide (NO) activity) [46]. In fact, infertile hydatid cysts are correlated with higher levels of NO, and it has been proposed that NO-producing immune cells are unable to penetrate the physical barrier imposed by the laminated layer [47], however, as seen in Fig 5D, this is a possibility.

The production of both the laminated layer and protoscoleces is a major metabolic activity of the germinal layer [44]. Our results show that cattle palisading macrophages are found surrounding infertile hydatid cysts, and are able to disorganize the laminated layer and infiltrate between the layers of this structure. The hydatid cyst will continue to grow as long as there is a steady production of laminated layer instead of protoscoleces, maintaining its infertility; if the balance is shifted towards the host immune cells, they can reach the germinal layer with the later destruction of the metacestode.

In conclusion, fertile hydatid cysts with mostly viable protoscoleces have adventitial layers with scar tissue, which means that the immune regulation molecules that the parasite secretes probably are aimed at triggering inflammation resolution in the adventitial layer. In cattle this event is rare, with a granulomatous immune response associated with low protoscolex viability, laminated layer disorganization and consequential immune cell infiltration.

## Supporting information

S1 Fig Laminated layer interaction with the adventitial layer. A) Laminated layer disorganization (black arrow) with complete sections of laminated layer in the middle of the adventitial layer, surrounded by inflammatory cells (green arrow). B) The adventitial layer infiltration between the different layers of the laminated layer makes it difficult to distinguish the two of them (red arrow). Stained with H&E. Size bar: A) 100 μm B) 50 μm.
(JPG)

S2 Fig Infertile hydatid cysts have host cells infiltrating the germinal layer. Most of the infertile cysts without detachment of the laminated layer (LL) have the presence of host immune cells (arrows) infiltrating the inner chamber of the cyst. Size bar 50 μm.
(JPG)

S1 Table Complete laminated layer measures (μm) of fertile, infertile and small bovine hydatid cysts, from both liver and lungs. (XLSX)

S2 Table Inflammation scores of fertile, infertile and small bovine hydatid cysts, from both liver and lungs. Sheet one has the raw data of each sample. Sheet two shows the sample proportion for each inflammatory score value. Sheet three has dichotomized data used for statistical analysis.
(XLSX)
