# Supplementary figures and images for: New insights of the local immune response against both fertile and infertile hydatid cysts
Source: PLoS One. 2019 Jan 30;14(1):e0211542. doi: 10.1371/journal.pone.0211542 (PMC6353198; doi:10.1371/journal.pone.0211542)

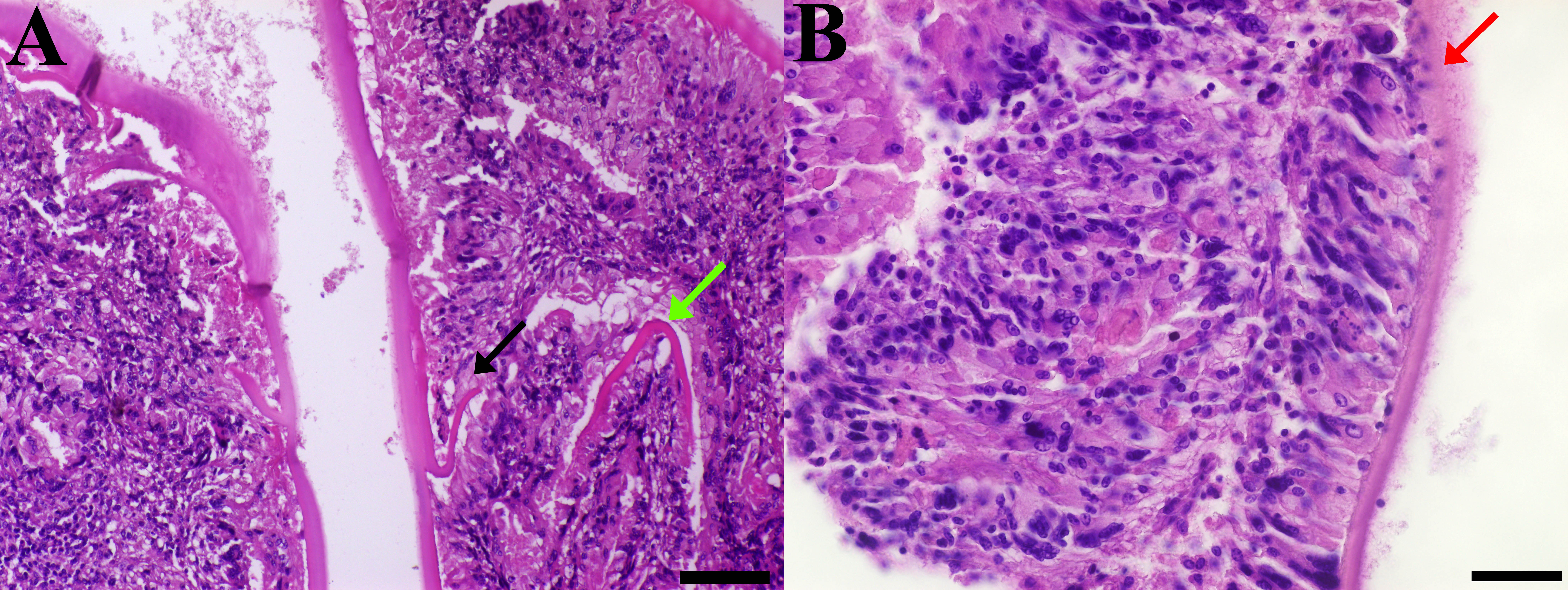

Supplement: S1 Fig — A) Laminated layer disorganization (black arrow) with complete sections of laminated layer in the middle of the adventitial layer, surrounded by inflammatory cells (green arrow). B) The adventitial layer infiltration between the different layers of the laminated layer makes it difficult to distinguish the two of them (red arrow). Stained with H&E. Size bar: A) 100 μm B) 50 μm. (JPG) [file pone.0211542.s001.jpg]

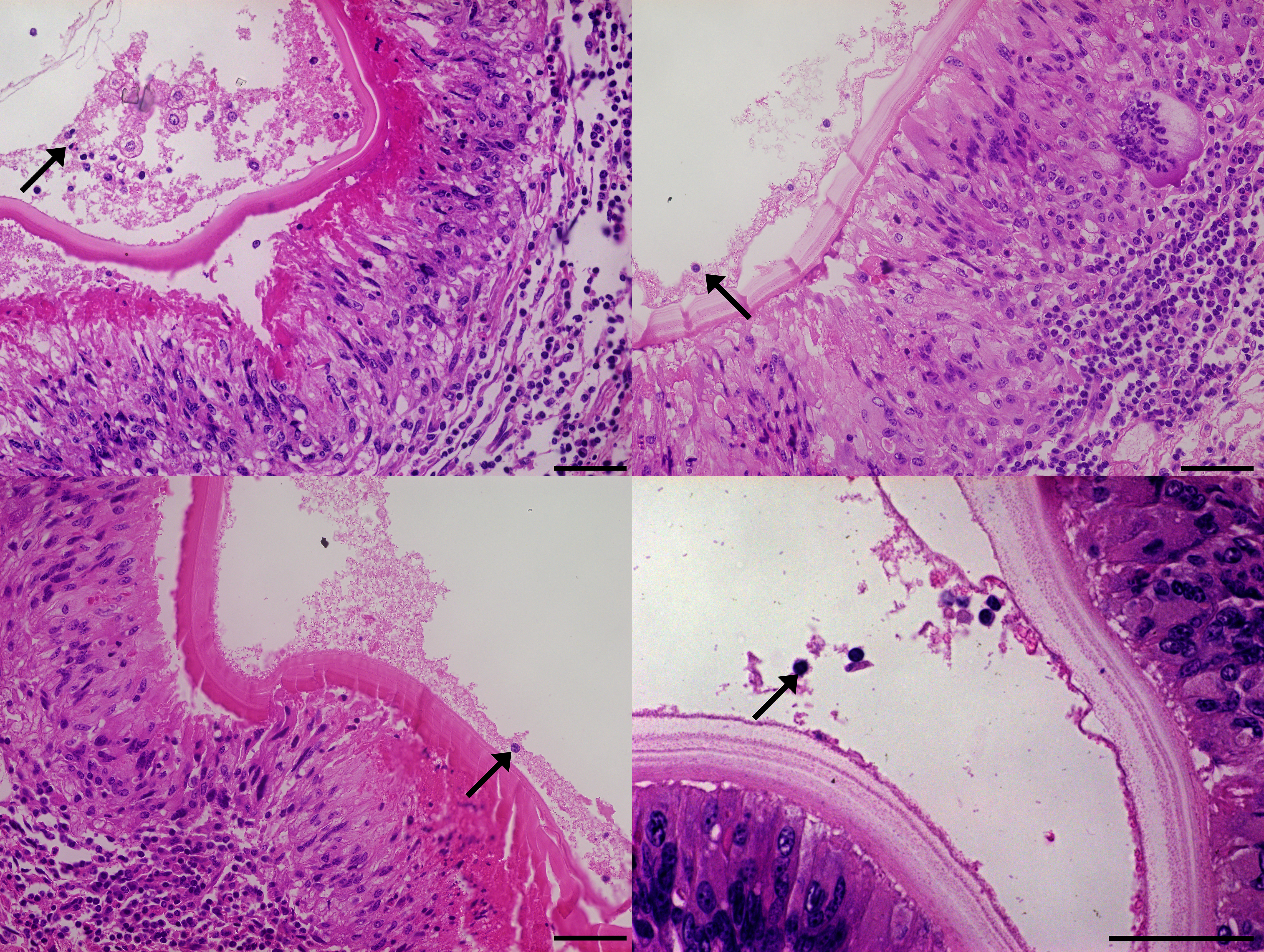

Supplement: S2 Fig — Most of the infertile cysts without detachment of the laminated layer (LL) have the presence of host immune cells (arrows) infiltrating the inner chamber of the cyst. Size bar 50 μm. (JPG) [file pone.0211542.s002.jpg]
